# Giant Aneurysm of The Non-Coronary Sinus of Valsalva

**DOI:** 10.21470/1678-9741-2019-0054

**Published:** 2020

**Authors:** Murat Ugurlucan, Yahya Yildiz, Ekrem Guler, Mustafa Ozer Ulukan, Didem Melis Oztas, Emin Can Ata, Aydin Kahraman, Korhan Erkanli, Halil Turkoglu

**Affiliations:** 1 Department of Cardiovascular Surgery, Istanbul Medipol University Medical Faculty, Istanbul, Turkey.; 2 Department of Anesthesia, Istanbul Medipol University Medical Faculty, Istanbul, Turkey.; 3 Department of Cardiology, Istanbul Medipol University Medical Faculty, Istanbul, Turkey.; 4 Bagcilar Training and Research Hospital, Cardiovascular Surgery Clinic, Istanbul, Turkey.

**Keywords:** Sinus of Valsalva, Aneurysm, Dilatation, Pathologic, Aorta, Heart Defects, Congenital, Connective Tissue

## Abstract

Aneurysms of the sinuses of Valsalva are defined as dilatation of the aortic root region between the aortic annulus and the sinotubular junction. Isolated aneurysms of the sinus of Valsalva are rare cardiovascular pathologies. They may be congenital, especially secondary to connective tissue disorders or in conjunction with congenital cardiac defects, or acquired such as secondary to infections or trauma. Small sized aneurysm without rupture in asymptomatic patients may be followed; however, latter cases require intervention and surgery is the gold standard treatment modality.

In this report, a 41-year-old male patient was reported with giant aneurysm of the non-coronary sinus of Valsalva whom underwent aortic root sparing surgical aortic sinus of Valsalva reconstruction.

**Table:** 

Abbreviations, acronyms & symbols	
CT	= Computerized tomography
ECHO	= Echocardiography

## INTRODUCTION

Aortic sinuses are sacs providing aortic opening during systole without occlusion of the coronary arteries. There are three aortic sinuses namely; right, left and non-coronary sinuses of Valsalva in accordance with the corresponding coronary artery. Although very rare, enlargement and aneurysm formation at the sinuses of Valsalva may occur^[1]^. Aneurysms may be confined to any of the sinuses of Valsalva. They may occur either congenitally, either in the course of congenital cardiac defects or secondary to genetic connective tissue disorders or secondary to various acquired etiologies^[2-4]^. Small aneurysms without rupture in asymptomatic patients may be followed; however, certain latter cases require intervention^[2,4]^.

In this report, a surgical treatment of a giant aneurysm of the non-coronary sinus of Valsalva with aortic root preservation is described.

### Case Report

**Fig. 1:**
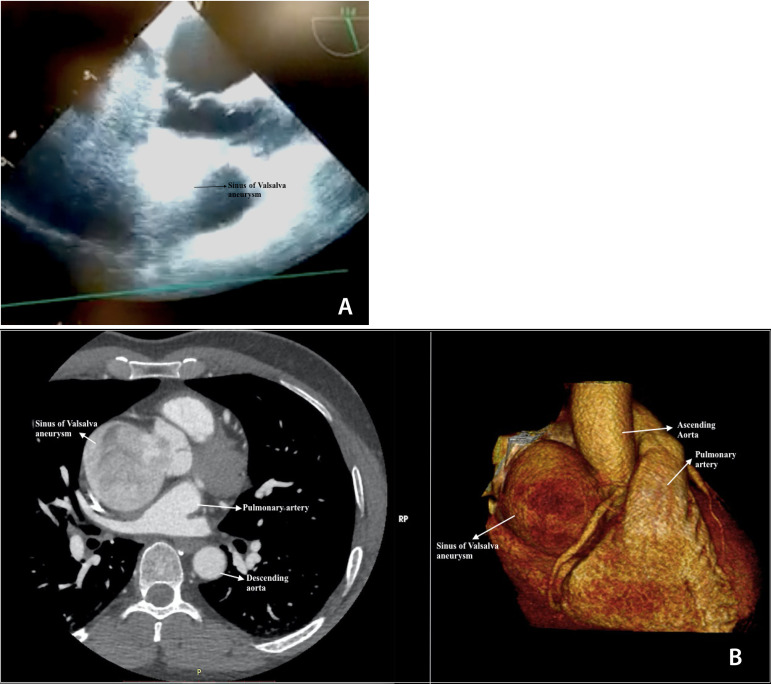
A) Echocardiography indicates dilatation of the non-coronary sinus; B) Computer tomography shows the aneurysm of the non-coronary sinus of Valsalva.

A 41-year-old male patient without a history of known health problems presented to the clinic with sudden onset chest pain radiating to the neck and back. He was found hypertensive with blood pressure of 210/90mmHg. Electrocardiogram and blood analysis were normal. Echocardiography (ECHO) indicated mild left ventricular hypertrophy, normal myocardial contractions, no extra valvular pathologies other than minimal aortic insufficiency; however, hugely dilated non-coronary sinus of Valsalva (Figure 1A). Computerized tomography (CT) revealed 6.5cm in diameter aneurysm of the non-coronary sinus of Valsalva compressing the right atrium and superior vena cava (Figure 1B).

**Fig. 2:**
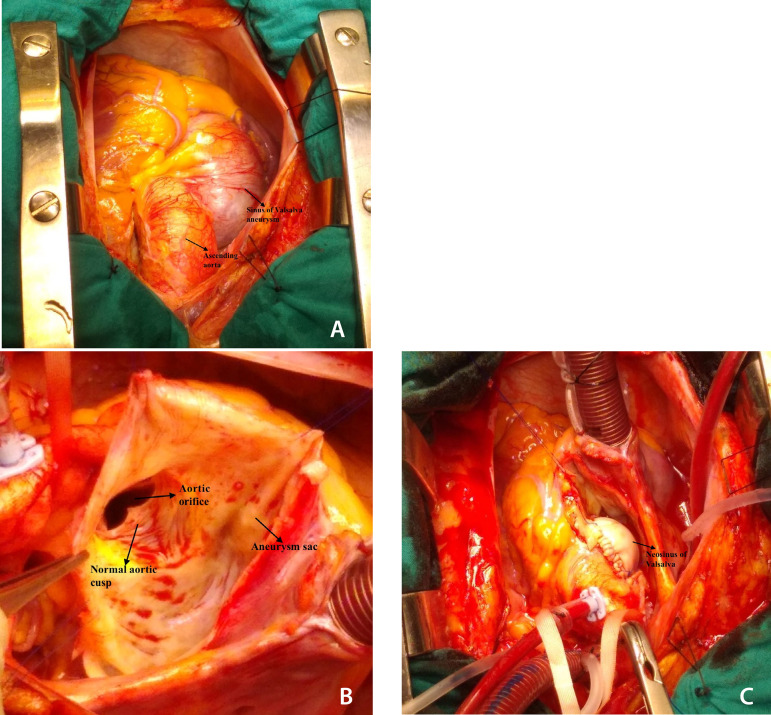
A) Perioperative view of the aneurysm; B) Aneurysm sac opened, showing normal aortic cusps; C) Biologic patch repair of the aneurysm of the non-coronary sinus of Valsalva.

Surgical treatment was offered to the patient and he was operated following his consent. Operation was started through full median sternotomy. Giant aneurysm bulging to the right was seen after opening the pericardium (Figures 2A and 2B). Aortic and two-stage venous cannulations were performed and cardiopulmonary bypass was initiated. Aorta was cross-clamped and heart was arrested with isothermic blood cardioplegia. The aneurysm was opened. The aortic valves were inspected and they were normal with normal coaptation. A neo-non-coronary sinus was created with a double layer xenograft pericardial patch (Edwards Lifesciences Corporation, Irvine, California, ABD) sutured to between the annulus and the ascending aorta (Figure 2C). Samples were obtained both from the ascending aorta and the dilated sinus for microbiologic and histopathologic examination. The remained aneurysm tissue was closed over the neo sinus. Patient was weaned off cardiopulmonary bypass without inotropic support and control treansesophageal ECHO showed no aortic regurgitation with normal myocardial functions. Cross clamp and cardiopulmonary bypass times were 17 minutes and 31 minutes, respectively. Patient was extubated on the second postoperative hour and total intensive care unit and hospital stays were 16 hours and 5 days, respectively.

Culture of the resected material resulted sterile and histopathologic examination indicated significant elastic fiber loss and increased connective tissue at the resected aneurysm and minimal fragmentation at the elastic fibers at the normal aortic wall. He has been followed asymptomatic with normal myocardial contractions and valvular functions without any regurgitation or dilatation at the patch region or any other parts of the aorta more than six months.

## DISCUSSION

Aneurysms of the Valsalva sinuses may be defined as enlargement of the aortic root region between the aortic annulus and the sinotubular junction^[1]^. Diameters of the sinuses varies by age and gender; however, accepted upper limits adjusted for body surface area in men is 4 cm and 3.6 cm in women^[2]^. Anatomically the left coronary sinus is in proximity with the anterior leaflet of the mitral wall and the left ventricular free wall. The right coronary sinus is above the interventricular septum and right ventricle. The non-coronary sinus lies above the interventricular septum, slightly anterior mitral leaflet and freely over the transverse sinus^[3]^.

Most of the cases are congenital and secondary to connective tissue disorders such as Marfan’s syndrome, Ehler-Danlos syndrome or various others; or may be in case of bicuspid aortic valve. Acquired cases may result from infectious agents affecting the sinuses such as bacterial endocarditis, syphilitic or tuberculous infections. Chest trauma, iatrogenic causes, or medial necrosis secondary to atherosclerosis are various other acquired etiologies. The estimated incidence of sinus of Valsalva aneurysms is 0.09% based on autopsy series^[2]^. The incidence is increased in case of congenital etiology and ranges between 0.1-3.5%^[5]^. The pathology affects the right coronary sinus, non-coronary sinus and the left coronary sinus with a decreasing frequency. Males are four times more frequently affected than females^[2]^. Our patient was a 41-year-old male who was diagnosed with 6.5cm in diameter aneurysm of the non-coronary sinus of Valsalva. He did not define a particular chest trauma and did not have any congenital cardiac defects or collagen vascular pathologies which may lead to the condition. The histopathologic examination of the excised sinus material was negative for infections and specific connective tissue disorders revealing significant elastic fiber loss and increased connective tissue at the resected aneurysm and minimal fragmentation at the elastic fibers at the normal aortic wall.

Most of the patients with aneurysm of sinus of Valsalva are usually asymptomatic or may have non-specific symptoms such as dyspnea, palpitations and chest pain. Other symptoms may be arrhythmia, heart block, thrombus formation in the aneurysm sac and leading to thromboembolic complications, coronary compression leading to ischemic heart disease or very frequently aortic regurgitation. Rupture of the right and non-coronary sinus aneurysms usually occurs into the right atrium or ventricle and leads to left to right shunt and right heart failure. Patients with ruptured aneurysms frequently present with acute onset dyspnea, exercise intolerance and congestive heart failure^[3]^. Our patient presented to the clinic with acute onset chest pain; however, which was more suggestive for a possible aortic dissection or myocardial infarction. He was diagnosed incidentally with giant aneurysm of the non-coronary sinus of Valsalva.

Echocardiography gives information about the sizes of the aortic sinuses as well as valvular pathology if present^[3]^. Cardiac magnetic resonance imaging is accepted as the gold standard diagnostic tool; however, nor required in most of the cases. Conventional coronary angiography may be needed in high risk patients for coronary atherosclerosis; otherwise, modern computed tomography angiography clearly presents the coronary pathologies in the current era^[2]^. Echocardiography was preferred as the initial examination tool and the pathology was confirmed with CT in our particular case.

Surgery is the gold standard treatment method for the aneurysms of the sinus of Valsalva, despite promising outcomes with endovascular closing devices in case of rupture^[2]^. Medical treatment is initiated for the stabilization of the patients with rupture and heart failure until surgery. Patients should be rapidly evaluated for treatment in case of aneurysm rupture, since rupture may lead to acute onset congestive heart failure due to left to right shunt^[5]^. Surgery is also indicated in case of ventricular septal defect or aortic regurgitation. Other indications for surgery rely on the size of the aneurysms and surgery is indicated for aneurysms larger than 5.5 cm, 5 cm in case of bicuspid aortic valve, 4.5 cm in case of connective tissue disorders and in patients with an aneurysm growth rate more than 0.5 cm/year^[2,4]^. Medical treatment is also required against the etiology of the aneurysm such as prophylaxis for infective endocarditis, vasculitis syndromes, hypertension, rhythm disturbances and ischemic heart disease^[4]^. The maximum diameter of the sinus Valsalva aneurysm in our patient was 6.5 cm and we offered surgical correction of the pathology. During surgery, we observed the wall of the aneurysm was very thin.

Aim of surgical treatment is to preserve the aortic root geometry with normal aortic valve functions^[6]^. Primary repair may be applied for small aneurysms; however, resection and patch repair are preferred in bigger cases. Since the disease is accompanied by aortic regurgitation in 30-50% of the cases, aortic valve replacement rate is not low in certain cases. Additional pathologies are also treated at the time of surgery^[2,3]^. Our patient had minimal aortic regurgitation detected by ECHO preoperatively. Perioperatively, at inspection and water test, he had good functioning tri-cusp aortic valve and valve sparing aortic root preservation was the chosen method. After resection of the dilated sinus of Valsalva, the non-coronary sinus of Valsalva was reconstructed with a xenograft pericardial patch. The control transesophageal ECHO showed no aortic regurgitation with normal myocardial functions.

In conclusion, sinus of Valsalva aneurysms is rare and treatment is indicated in big size aneurysms to prevent complications and death. Surgery with an aim to preserve aortic root geometry and valvular functions are the main goals of treatment; however, when not possible, aortic root replacement has to be inevitable.

**Table:** 

Author's roles & responsibilities	
MU	Substantial contributions to the conception or design of the work; or the acquisition, analysis, or interpretation of data for the work; drafting the work or revising it critically for important intellectual content; agreement to be accountable for all aspects of the work in ensuring that questions related to the accuracy or integrity of any part of the work are appropriately investigated and resolved; final approval of the version to be published
YY	Substantial contributions to the conception or design of the work; or the acquisition, analysis, or interpretation of data for the work; agreement to be accountable for all aspects of the work in ensuring that questions related to the accuracy or integrity of any part of the work are appropriately investigated and resolved; final approval of the version to be published
EG	Substantial contributions to the conception or design of the work; or the acquisition, analysis, or interpretation of data for the work; agreement to be accountable for all aspects of the work in ensuring that questions related to the accuracy or integrity of any part of the work are appropriately investigated and resolved; final approval of the version to be published
MOU	Substantial contributions to the conception or design of the work; or the acquisition, analysis, or interpretation of data for the work; final approval of the version to be published
DMO	Drafting the work or revising it critically for important intellectual content; agreement to be accountable for all aspects of the work in ensuring that questions related to the accuracy or integrity of any part of the work are appropriately investigated and resolved; final approval of the version to be published
ECA	Substantial contributions to the conception or design of the work; or the acquisition, analysis, or interpretation of data for the work; agreement to be accountable for all aspects of the work in ensuring that questions related to the accuracy or integrity of any part of the work are appropriately investigated and resolved; final approval of the version to be published
AK	Drafting the work or revising it critically for important intellectual content; agreement to be accountable for all aspects of the work in ensuring that questions related to the accuracy or integrity of any part of the work are appropriately investigated and resolved; final approval of the version to be published
KE	Drafting the work or revising it critically for important intellectual content; agreement to be accountable for all aspects of the work in ensuring that questions related to the accuracy or integrity of any part of the work are appropriately investigated and resolved; final approval of the version to be published
HT	Drafting the work or revising it critically for important intellectual content; agreement to be accountable for all aspects of the work in ensuring that questions related to the accuracy or integrity of any part of the work are appropriately investigated and resolved; final approval of the version to be published
